# Study on a performance matching method of wind rotor and generator based on energy transfer

**DOI:** 10.1371/journal.pone.0294504

**Published:** 2023-11-22

**Authors:** Minghui Ma, Lei Song, Yabin Jia, Zongxiao Yang

**Affiliations:** 1 School of Mechatronics Engineering, Henan University of Science and Technology, Henan, Luoyang, China; 2 Henan Engineering Laboratory of Wind Power Systems, Henan, Luoyang, China; Public Library of Science, UNITED KINGDOM

## Abstract

Wind power systems are a promising form of energy supply. At present, most of the studies focuses on the performance of individual components such as wind rotors or generators, and the overall output effect of wind power system is determined by the characteristics of wind rotor and generator and their combined characteristics. However, the evaluation of the overall output characteristics of the system is rarely considered. In order to investigate the overall output of the system quickly, a performance matching method of wind rotor and generator based on energy transfer is proposed in this paper. Based on the series operating characteristics of the wind power system model, the energy transformation process of the wind rotor, generator and the whole system are unified described by energy transfer. On the premise that the performance of wind rotor and generator is known, the transfer function model of each component is established, and on this basis, the transfer function model of the overall system is obtained. Then, the overall output effect of the system is analyzed and evaluated by this system transfer function model. The performance of the model is analyzed and compared by using a vertical axis wind power system coupling test bench and MATLAB/Simulink software. The results show that the error between the system output based on the theoretical model and the wind tunnel test is less than 6.5%, and the trend of the simulation and the test curve of the system output is consistent. Therefore, this method can be used to quickly predict the overall output performance of the wind turbine and generator on the premise that the performance of each component is known, without the need to connect each component to a wind power system for testing.

## 1. Introduction

As the fossil energies becoming drying up, wind energy has taken more and more attention by countries all over the world at present. However, wind power system is a complex energy conversion system composed of various mechanical, electrical and control components such as wind rotor, generator and controller, and the overall output performance depends on the combined influence of wind rotor, generator, etc. At present, the studies mainly focus on the performance of kinds of wind rotors or generators respectively, and their structures and performance have been deeply analyzed [1, 2]. At present, improving the performance of a wind rotor has been a significant focus of study [3]. To describe the developments in the design of electrical generators, it is necessary to look at the conversion system as a whole [4]. The interaction between these components also has a great influence on the overall output performance of a wind power system. If designers can quickly find a wind rotor and a generator with matching performance in existing products so as to quickly build a wind power system and analyze and forecast its performance, it will greatly improve the design efficiency and reduce the design cost for a wind power system. However, studies on the matching of the wind turbine and generator are rare and the matching problem is worthy of being discussed [5]. Therefore, it is a key problem to explore the analytical methods of the overall output model of a wind power system and the performance matching between components. This study explores the matching techniques that will predict the overall performance of a wind power system and improve the design efficiency.

Scholars have carried out continuous studies on blade design, generator selection and system performance of wind rotors. Bao et al. substituted the mechanism analysis method into the model construction of wind power system, and established the wind power system model with the mechanism model structure by combining with the identified model parameters [6]. Agustín et al. proposed to establish the state-space model of PMSG (Permanent Magnet Synchronous Generator) wind turbines, and realized a complete model of PMSG wind turbines connected to their control through full-size back-to-back converters by using the detailed model contained in the real-time digital emulator [7]. Besheer et al. used a T-S model (Takagi-Sugeno Models) to describe the nonlinear wind energy conversion system, and designed a fuzzy feedback controller with stable fuzzy model to describe the control requirements and the inherent nonlinear characteristics of the system [8]. Zhang et al. presented an approach for wind farm outgoing capability which considering the wind turbine’s output features and the allowable carrying current of transmission lines [9]. Diyoke derived a new approximate capacity factor equation for matching wind turbines to potential site for optimum yield and profitability [10]. Ali et al. proposed an analytical method to test the operating performance of horizontal axis wind turbine considering that the rotor was constrained by the torque-speed characteristics of coupled generator [11]. Barakati et al. built a complete wind power system model and measured the output characteristics of the system with instruments by simulating the output of the wind turbine through a controllable motor [12]. Shan et al. proposed a modeling method for wind power generation systems based on wind speed model, which can be used to study the momentum problems in wind power generation and the mechanical torque of the generator [13]. These studies show that wind power systems can be expressed and analyzed by constructing models. Aredjodoun et al. optimized the synchronous generator with permanent magnets, and ensuring mutual adaptation between the wind rotor and the optimized generator, so that the assembly was more attractive and more sensitive to the conversion of wind energy [14]. Xie et al. studied the matching characteristics of the wind turbine and generator of the small H-shaped vertical axis wind turbine [15]. Their results showed that the power characteristic curve and torque characteristic curve of the generator wind wheel were respectively overlap the best power curve and best torque of the generator, the matching characteristics of the small H-shaped vertical axis wind turbine rotor and generator are optimal. Pan et al. established the mathematical model of an identification model for a fixed-speed fan transmission system, and identified the undetermined parameters in the model through the Levenberg-Marquardt estimation method and compared them with the actual values [16]. Gordievsky presented MATLAB Simulink model of Vertical Axis Wind Turbine to demonstrate the functional components and simulation modes [17]. Chabaud proposed a novel approach for the modeling of rotor-integrated aerodynamic loads considering all the six degrees of freedom (including tangential components), and parameter identification methods were suggested also [18]. Elkington et al. proposed a 3rd-order doubly-fed induction generator electromotive force model, which with was similar to the commonly used 5rd-order wind turbine model [19]. Studies of Vlad et al. showed that the overall efficiency of low power wind turbines was closely related to generator performance, electrical power optimization and mechanical performance optimization [20]. Kao proposed to develop a matching program between wind turbine blades and generators to improve the efficiency of wind power systems by determining the optimal constant pressure mode for generators [5]. These studies provide ideas and referential methods for component modeling.

Transfer functions are often used to describe the relationship between the inputs and outputs of a system. Al-khamis compared the performances of the transfer function based model and the state space based model in Model predictive control design for Brushless doubly-fed induction generators, and the study demonstrated that using the transfer function to calculate the parameters of the MPC (Model Predictive Control) can eliminate the drawbacks of other design models [21]. Chen et al. analyzed VGS (Virtual Synchronous Generator) control based on transfer function, which can be used as the basis for the design of VSG transient and steady-state performance [22]. Chen et al. derived impedance model of doubly fed induction generator in sequence domain considering frequency coupling effect based on the complex vector modelling method [23]. Kuschke et al. rearranged and linearized the originally nonlinear behavior of the drive system covering turbine, permanent magnet synchronous generator, and obtained a compact transfer function description [24]. Cicenas et al. proposed real measurements of the aggregation approach to ensure the stability of the power system [25]. The proposed methodology was orientated toward obtaining transfer functions that were developed using the parametric identification models. Thus, the transfer function can describe the characteristics of kinds of complex systems.

At present, most of the studies focuses on the performance of individual components such as wind rotors or generators. However, the evaluation of the overall output characteristics of the system is rarely considered. Our work focuses on the ability to quickly predict the overall output performance of a wind power system, while the performance of its components such as wind rotor and generator are taken and without the need to be connected to the wind power system for testing. Based on ideas of scholars, a performance matching method for wind rotor and generator based on energy transfer is proposed in this paper. The energy transformation process of the wind rotor, generator and the system are described by energy unified based on the series operation characteristics of the wind power system physical model. The transfer function models of each component and the system are constructed, and the overall output effect is considered to investigated quickly by the model.

The remainder of this paper is structured as follows: Firstly, the construction of transfer function model for wind power system is described. Secondly, transfer functions of a wind rotor specimen and a generator specimen are described, and the overall transfer function for the wind power system is obtained. The output performance of the system is analyzed and estimated by this transfer function, and compared with that of the wind tunnel test. Finally, concluding remarks are presented in Section 4.

## 2. System model

### 2.1 Wind power system model

**Fig 1:**
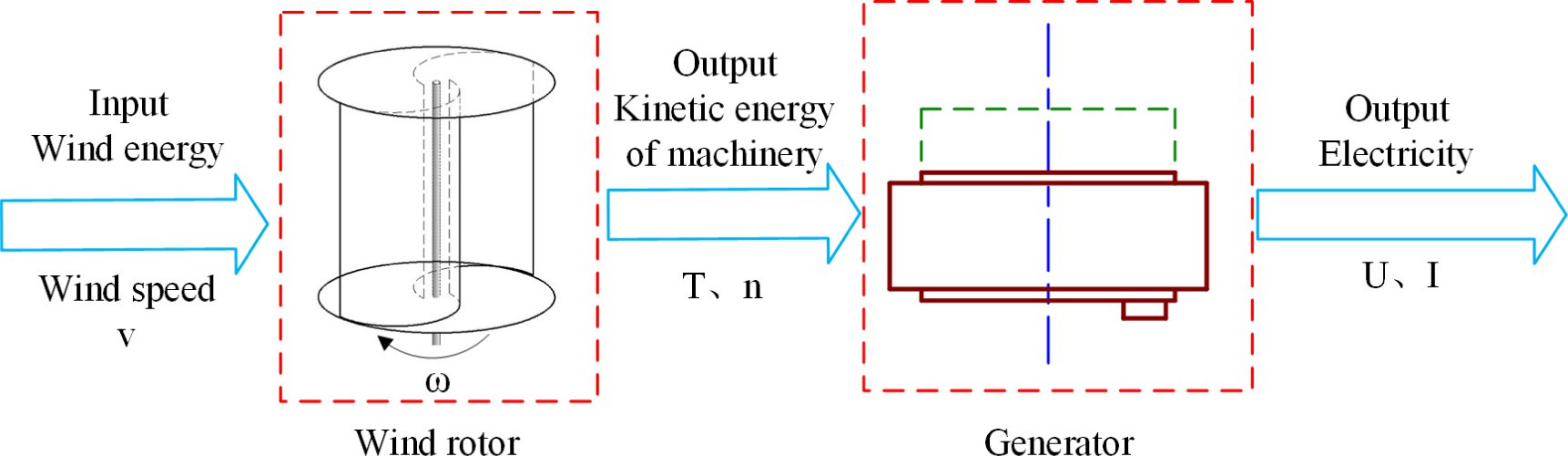
Physical model of wind power system. Wind drives the wind rotor rotating to convert wind energy into mechanical energy, and the generator connected with wind rotor also rotates to produce voltage and current.

The main components of a wind power system include wind rotor, generator, controller, etc., among which the key components for the conversion of wind energy into electric energy are wind rotor and generator. Driven by the wind, the wind rotor rotates to convert wind energy into mechanical energy. The generator driven by the wind rotor rotates to produce voltage and current. The model is shown as Fig 1.

Generally, wind rotors and generators have their own characteristic curves. The input parameter of a wind rotor is wind speed usually, and outputs are torque and speed. The input parameter of a generator is the rotor speed, and the outputs are current and voltage. Since they have different structures and working parameters, their curves of operating characteristics are also different. Therefore, when a wind rotor is connected to a generator to form a wind turbine integrated system, the characteristic curves of the wind rotor or generator are no longer applicable to the whole system. The overall output performance of the system depends on the aerodynamic characteristics of the wind rotor, the electrical characteristics of the generator and their combination characteristics.

### 2.2 Construction of transfer function model for wind power system

As shown in Fig 1, when the structures of a wind rotor and a generator are determined, the overall output characteristics of the system are determined. The wind rotor is driven by the wind and its input *P*_*w*_ can be expressed as Eq 1. Wind energy is converted into rotary mechanical energy, and its output *P*_*m*_ can be expressed in Eq 2.


$$P_{w}=\frac{1}{2}\rho Av^{3}$$ (1)

Where, *ρ* is the air density, *A* is the cross-sectional area of the wind rotor, *v* is the wind speed.


$$P_{m}=\frac{Tn}{9550}$$ (2)

Where, *n* is the rotating speed r/min, and *T* is the torque Nm. *P*_*m*_ is measured in kilowatts. The value 9550 is the calculation factor.

Input of the generator is the output of the wind rotor. The output *P*_*e*_ of the generator is expressed as Eq 3.


$$P_{e}=\sqrt{3}U_{e}I_{e}\cos\varphi$$ (3)

Where, *U*_*e*_ is the output voltage, *I*_*e*_ is the output current, and *φ* is the potential angle.

Fig 1 shows that the energy of the wind power system flows through the wind rotor and generator in series. When the input and output can be described by a single parameter, transfer function model can be introduced to describe the energy transfer process of the system. Generally, the input and output corresponding to the transfer function *G*(*s*) are single factors. However, the input and output of each component often contain two or more variables, such as wind speed, rotating speed, torque, voltage and current. As the input and output of the system, energy can meet the requirements of the single factor model for the transfer function, thus the concept of energy transfer is introduced to establish the transfer function model of the wind power system. To simplify the analysis, the power variation is considered to describe the input-output relationship of the system.

#### 2.2.1 Transfer function model of wind rotor

**Fig 2:**
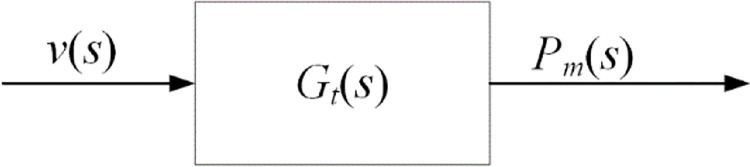
Block diagram of energy transfer of wind rotor. The wind speed v and power *P*_*m*_ are the input and output of the wind.

According to Eq 1, when the shape and size of a wind rotor are determined, the wind speed *v* determines the power input *P*_*w*_. The wind speed v and power *P*_*m*_ are selected as the input and output of the wind rotor. The energy transfer block diagram of wind turbine is shown in Fig 2.

As shown in Fig 2, the transfer function *G*_*t*_(*s*) of the wind rotor is shown in Eq 4.


$$G_{t}(s)=\frac{P_{m}(s)}{v(s)}$$ (4)

#### 2.2.2 Transfer function model of generator

**Fig 3:**
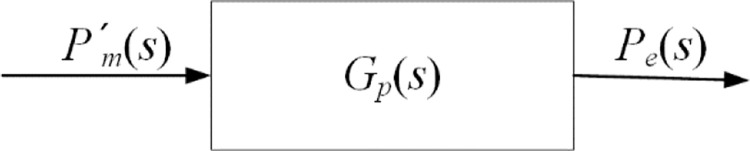
Block diagram of energy transfer of generator. *P’*_*m*_ and *P*_*e*_ are input and output of the generator.

The input power *P’*_*m*_ of the generator is the output power of the wind rotor. Through the output voltage *U*_*e*_ and current *I*_*e*_ of the generator under different rotating speed *n*, the output power *P*_*e*_ can be calculated as Eq 3. *P’*_*m*_ and *P*_*e*_ are selected as input and output of the generator. The energy transfer block diagram of the generator is shown in Fig 3.

As shown in Fig 3, the transfer function of the generator is shown in Eq 5.


$$G_{p}(s)=\frac{P_{e}(s)}{P_{m}^{'}(s)}$$ (5)

#### 2.2.3 Transfer function model of wind power system

**Fig 4:**
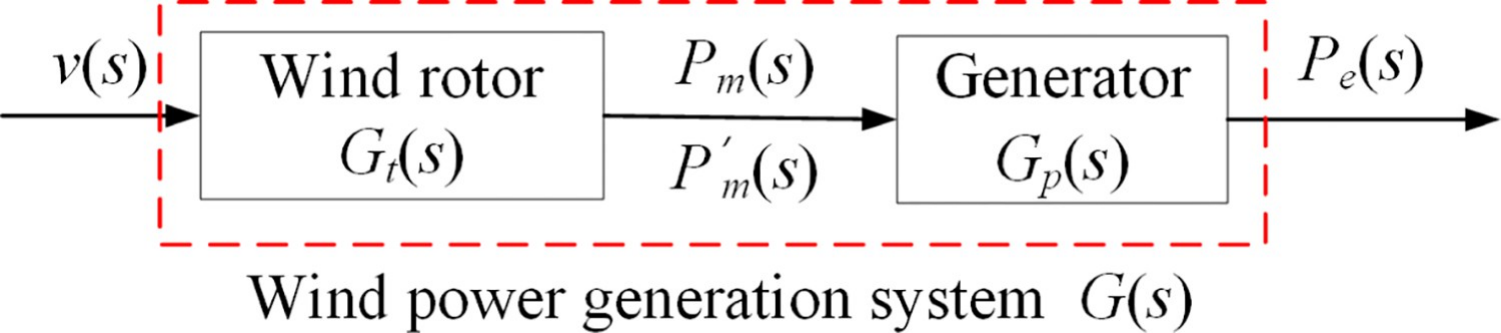
Block diagram of energy transfer of wind power system.

For a wind power system composed of a wind rotor and a generator, the internal transfer relationship can be simplified as the structure shown in Fig 4.

Where, *v*(*s*) and *P*_*m*_(*s*) are input and output of wind rotor *G*_*t*_(*s*) respectively, and *P’*_*m*_ (*s*) and *P*_*e*_(*s*) are input and output of generator *G*_*p*_(*s*) respectively. (Merge with the next segment)

The total output *P*_*e*_(*s*) of the system can be obtained in Eq 8.


$$P_{e}(s)=G_{t}(s)G_{p}(s)v(s)$$ (6)

Fig 4 indicates that the wind power system meets the series characteristics of the transfer function. Therefore, for a series-connected wind power system, its total transfer function is equivalent to the product of transfer functions of components in the system. *G*(*s*) is the equivalent transfer function of the wind power system connected in series, as shown in Eq 9.


$$G(s)=G_{t}(s)G_{p}(s)$$ (7)

According to Eq 9, when a wind rotor and a generator with different structures and characteristics are used in the wind power system, the overall output performance of the system is also different. Thus, the transfer functions of different components can be used to quickly match and predict the overall performance of the system.

## 3. Test results and discussions

Output characteristics of the wind power system are determined when the structures of each component of the system have been designed. In order to investigate the accuracy of the transfer function model shown in Eq 9, two specimens of a wind rotor and a generator with definite structures are selected and their performance parameters are obtained through the tests firstly. Then the transfer function models of the two specimens and the system are obtained by using MATLAB/Simulink software. Finally, the analysis on system performance matching and evaluation for transfer function model are carried out.

### 3.1 Identification of wind rotor transfer function model

#### 3.1.1 Wind rotor performance acquisition

**Fig 5:**
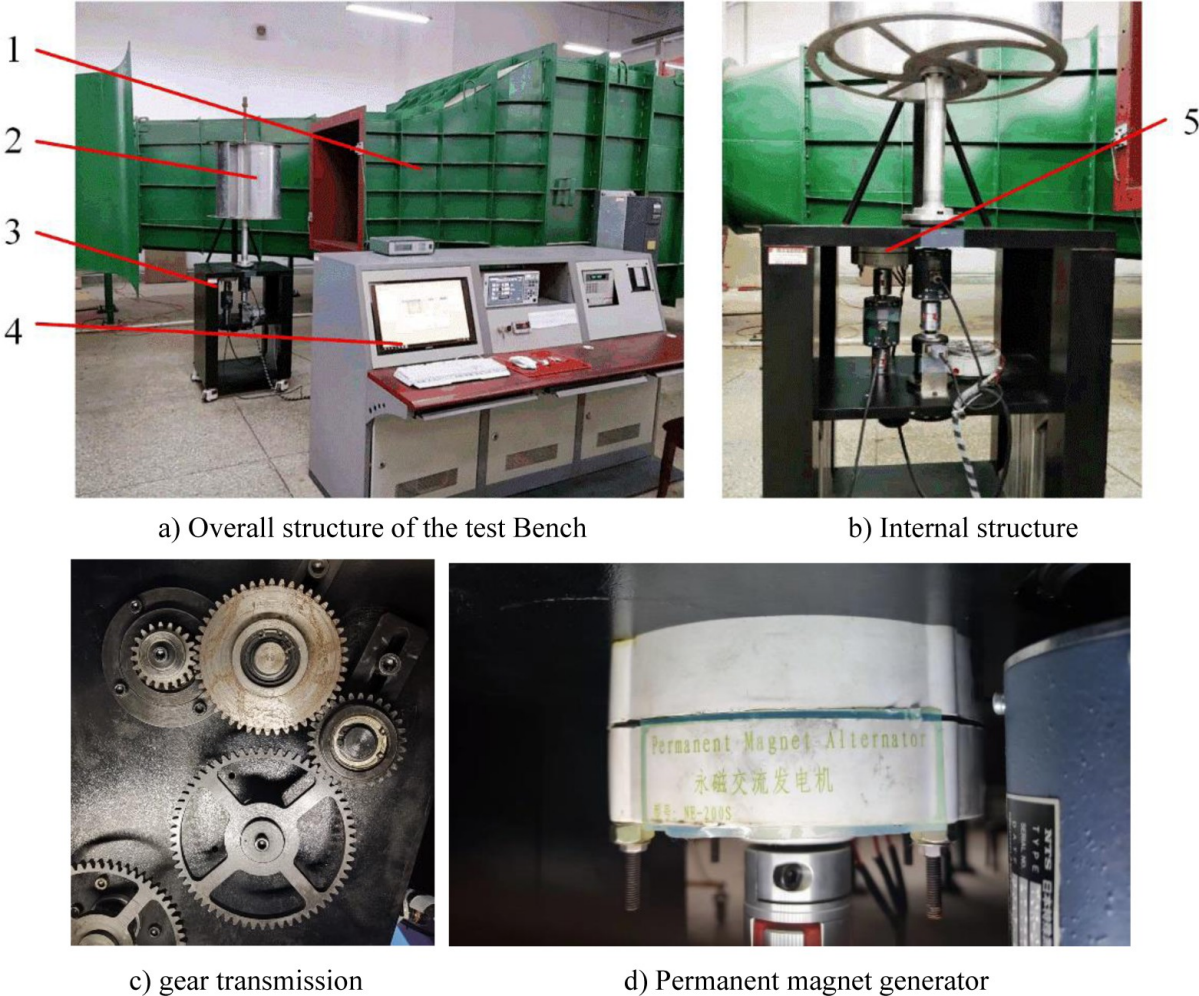
Vertical axis wind power system coupling test bench. a) Overall structure of the test Bench b) Internal structure c) gear transmission d) Permanent magnet generator. 1. Open-jet closed circuit wind tunnel 2. Wind rotor 3. test bench 4. Signal acquisition and analysis system 5. Generator.

A Savonius wind rotor with three blades is selected as the test specimen. The geometrical dimensions of the specimen are as follows: diameter is 450 mm, height is 450 mm, height-diameter ratio is 1.0, diameter of the central shaft is 45 mm, the overlap ratio is 0.256, and the specimen material is aluminum. A vertical axis wind power system coupling test bench is composed of open-jet closed circuit wind tunnel, wind rotor specimen bench, gear transmission system, outer rotor permanent magnet generator specimen, signal acquisition and analysis system and loading system. The test bench is shown in Fig 5. Fig 5A shows the overall structure of the test bench. Fig 5B shows the internal structure including generator and signal acquisition and analysis system. Fig 5C shows the gear increasing mechanism and the total growth ratio is 3. Fig 5D shows the permanent magnet generator specimen. Its model number is NE-200S. It is a three-phase permanent magnet alternator with rated power of 100W 200 W, rated voltage of 24 V and rated speed of 750 r/min.

**Fig 6:**
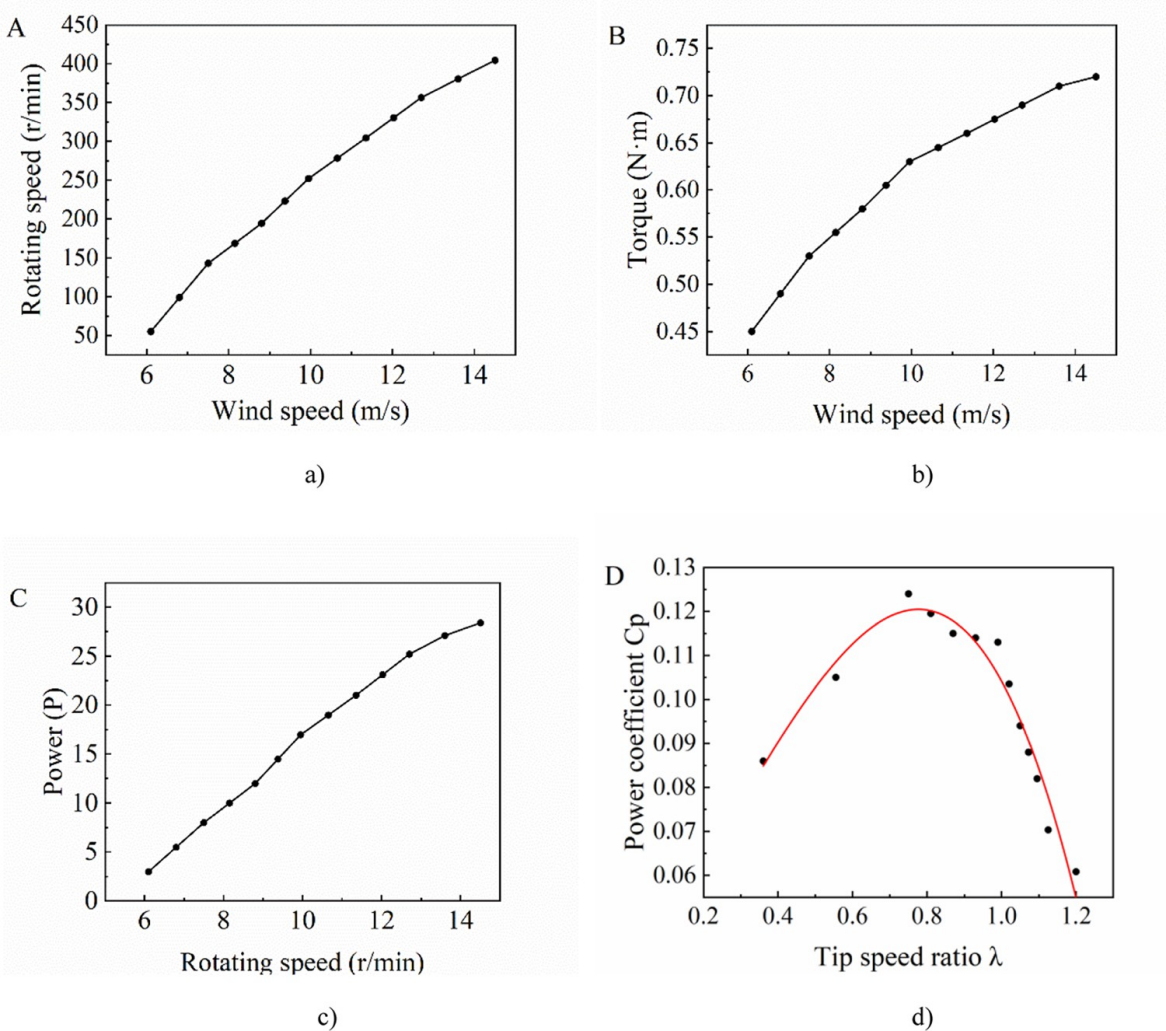
Output performance curves of the wind rotor. a) Wind speed vs rotating speed b) Wind speed vs Torque c) Rotating speed vs Power d) Tip speed ratio vs power coefficient.

According to the wind tunnel test, the main output performance curves of the wind turbine specimen are shown in Fig 6.

As shown in Fig 6A, the rotating speed increases while wind speed increases under the test conditions, and the relationship between them is approximately linear. From Fig 6B, the output torque increases with the increase of wind speed, and its increase rate decreases gradually. Fig 6C shows that the output power of the wind rotor increases while wind speed increases. Fig 6D shows that power coefficient *C*_*p*_ of the wind rotor increases with the increase of tip speed ratio *λ*, and then decreases after reaching a peak. The maximum *C*_*p*_ is 0.12, which is close to that of typical three-blades Savonius wind rotor, and the trend of power coefficient curve is roughly the same. Therefore, the specimen can be used as the test object of wind rotor model identification.

#### 3.1.2 Wind rotor model identification

The wind speed *v* and output power *P*_*m*_ are selected as the input and output of the wind turbine respectively. The System Identification module in MATLAB software is used to identify the transfer function model. The optimal transfer function model is obtained by adjusting the number of poles and zeros, as shown in Table 1. After inputting the poles and zero points, the fitting degree between the wind rotor transfer function curve and the test curve can be obtained in the Plant Identification Progress.

**Table 1:** The equations and the fitting degrees of each order model of the wind rotor.

Model	Pole	Zero	Equation	Fitting degree
*tf*1	1	0	$\frac{12.15}{s+0.7221}$	95.48%
*tf*2	1	1	$\frac{-2.282s+17.2}{s+1.228}$	96.03%
*tf*3	2	0	$\frac{118.3}{s^{2}+7.703s+9.029}$	96.42%
*tf*4	2	1	$\frac{-7.208s+144.2}{s^{2}+8.657s+11.18}$	96.42%
*tf*5	2	2	$\frac{-4.519s^{2}-223.4s+965.8}{s^{2}+39.78s+79.54}$	96.24%

Notes for Table 1: Fitting degree, the fitting degree between the wind rotor transfer function curve and the test curve

**Fig 7:**
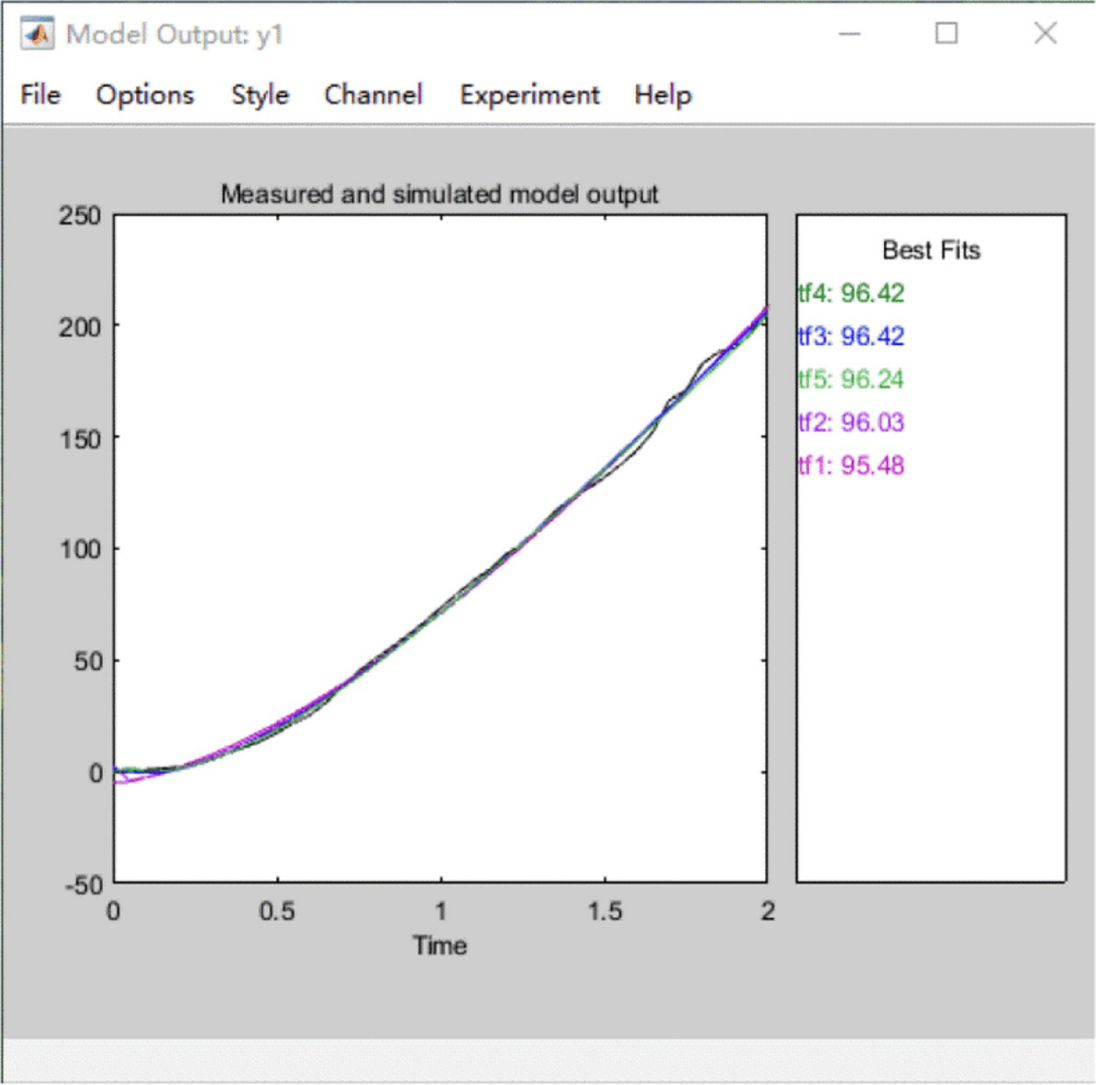
Step response curves of each model of wind rotor. *tf*1 to *tf*5 are the step response curves of five groups of transfer function models.

By adjusting the number of poles and zeros of the transfer function, five different groups transfer function model equations and the fitting degrees between simulation and test data are obtained. The fitting degree of *tf*3 and *tf*4 equations is higher than the other three equations. The step response curves of each order transfer function model are shown in Fig 7, in which the order of *tf*4 is lower and the step response speed is faster than that of *tf*3. Therefore, the transfer function of the wind rotor specimen is determined as *tf*4 in Tab.1 and is shown in Eq 8.


$$G_{t}(s)=\frac{-7.208s+144.2}{s^{2}+8.657s+11.18}$$ (8)

### 3.2 Transfer function model identification of generator

#### 3.2.1 Generator performance acquisition

**Fig 8:**
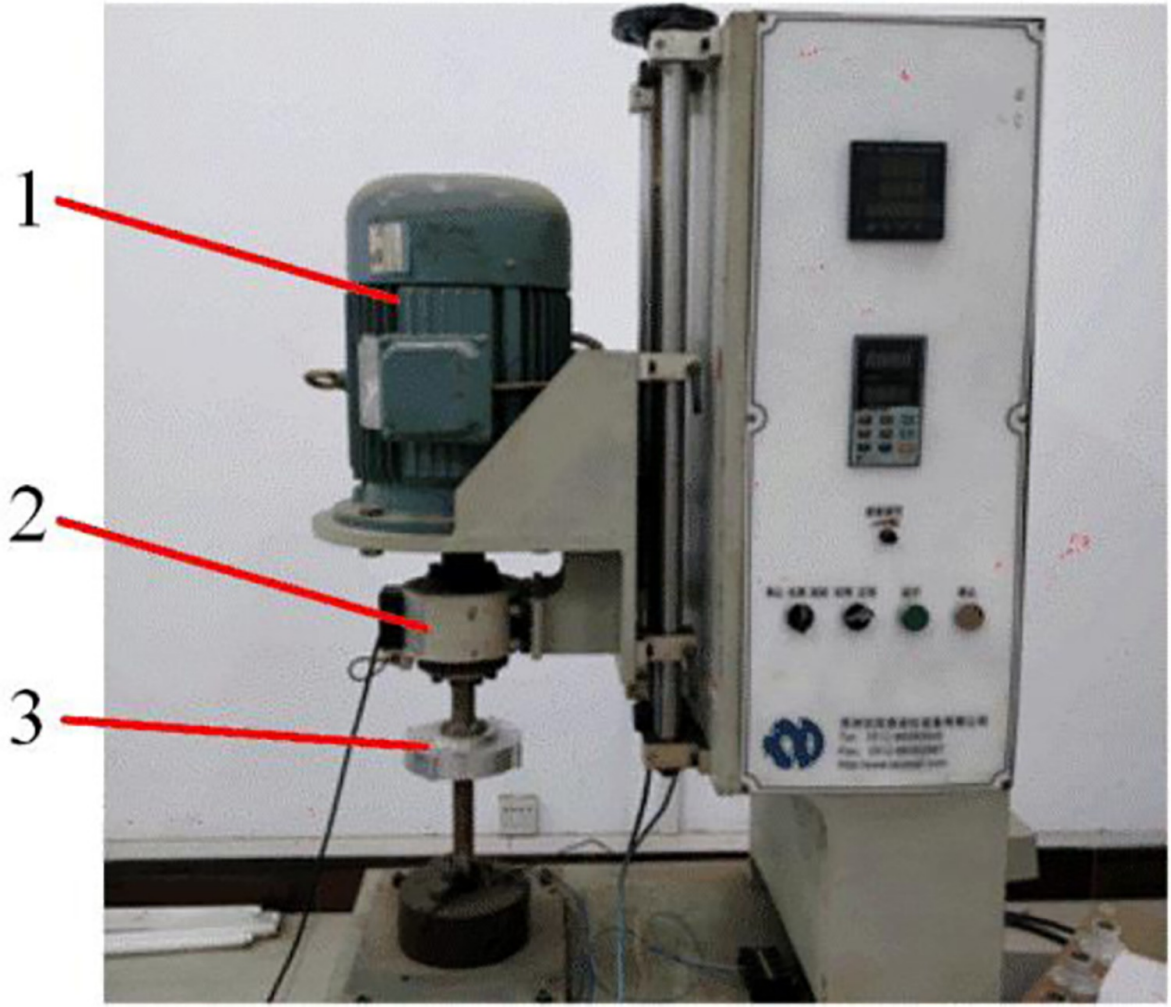
The generator performance inspection test bench. 1. Electric motor, Since the output of the generator is three-phase alternating current, the load circuit consists of a rectifier bridge and a set of light bulbs. 2. Tachometer, The speed of the motor is adjusted by the frequency converter and measured by the speed sensor. 3. Generator, The output voltage, current and power of the generator at different speeds are obtained by the power analyzer.

**Fig 9:**
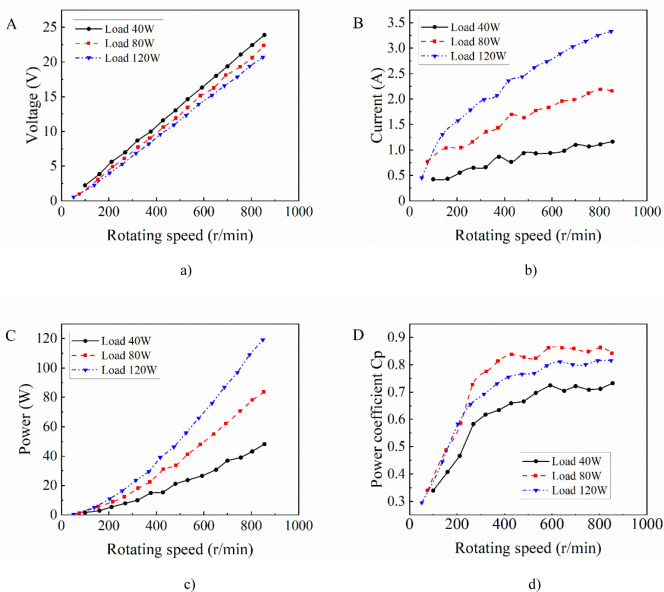
The output performance curves of the generator. a) Rotating speed vs Voltage. b) Rotating speed vs Current. c) Rotating speed vs power. d) Rotating speed vs power coefficient.

The tested generator specimen is a three-phase permanent magnet alternator with rated power of 200 W, rated voltage of 24 V and rated speed of 750 r/min. In order to study matching relation between generator and wind rotor, the generator that doesn’t match the performance of the wind rotor is selected. The output characteristics of the generator are tested on a generator performance inspection test bench, as shown in Fig 8. Since the output of the generator is three-phase alternating current, the load circuit consists of a rectifier bridge and a set of light bulbs. The speed of the motor is adjusted by the frequency converter and measured by the speed sensor. The output voltage, current and power of the generator at different speeds are obtained by the power analyzer. Fig 9 shows the output performance curves of the generator specimen.

As shown in Fig 9A, the output voltage increases linearly with the rotating speed under the same load within the test range. For different loads, the output voltage of the generator driving a small load is higher than that of a large load at the same speed, and the voltage difference is more obvious with the increase of the speed. Therefore, the degree of load has a great influence on output voltage of the generator. As shown in Fig 9B, the higher the rotational speed, the larger the output current of the generator under the same load condition. When the generator keeps at the same speed, the output currents under three loads show a certain multiple relationship with the increase of load multiple. As shown in Fig 9C, when the generator reaches the rated speed, the output power under three loads is 39.04 W, 70.57 W and 96.9 W respectively, accounting for 39.04%, 70.57% and 96.9% of the rated power. The results show that the load values of 40 W and 80W are too small, and the output power value of the generator is far from reaching the rated power at rated speed. However, the output power of the load 120 W is close to the rated power value at the rated speed, and the error is only 3.1%. Thus, the load 120 W can bring the generator closer to the optimal operating condition compared with the other two. This also indicates that too small or too large loads will reduce the output performance of the generator, and the best output performance of the generator can only be achieved by matching the appropriate load. Fig 9D shows that the power coefficients of the generator increase first and then tends to be stable with the increase of the rotational speed, and then has a downward trend. Rotating speed of the generator in the wind power system is provided by the wind rotor, however, the characteristic curves of them cannot achieve the optimal performance at the same speed, so the total output of the wind power system is their interactions.

#### 3.2.2 Generator model identification

The *P’*_*m*_ corresponding to the output power *P*_*m*_ of the wind rotor and *P*_*e*_ are selected as the input and output of the generator respectively. The system identification in MATLAB software is used to identify the transfer function model. The optimal transfer function model is obtained by adjusting the number of poles and zeros, five groups of different transfer function model equations of the generator and the fitting degrees of simulation and test data are obtained in Plant Identification Progress. The model of the generator’s transfer functions and its fitting degrees are shown in Table 2. It shows that the fitting degrees of *tf*13, *tf*14 and *tf*15 equations are too lower than the other two.

**Table 2:** Equations and fitting degrees of each order of generator.

Model	Pole	Zero	Equation	Fitting degree
*tf*11	1	0	$\frac{2.817}{s+2.253}$	95.06%
*tf*12	1	1	$\frac{0.3487s+0.8253}{s+0.1633}$	97.39%
*tf*13	2	0	$\frac{1245}{s^{2}+1430s+38.08}$	70.94%
*tf*14	2	1	$\frac{-0.9578s-8.192}{s^{2}+9.12s+5.159}$	-93.40%
*tf*15	2	2	$\frac{0.1332s^{2}+0.4851s+0.3911}{s^{2}+1.498s+0.3075}$	58.61%

**Fig 10:**
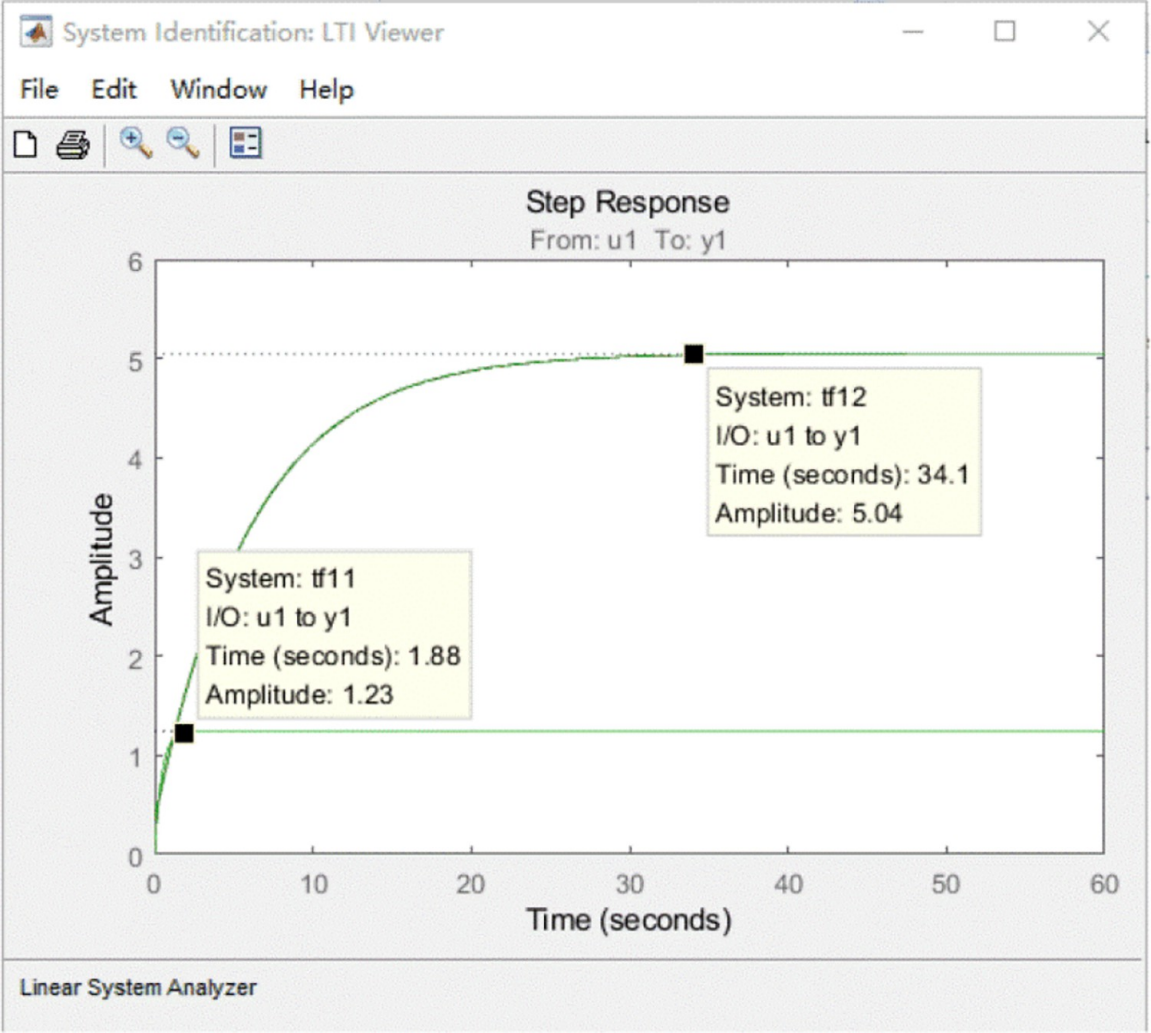
Step response curve of each model of generator. The order of *tf*11 is lower and the step response speed is faster than *tf*12.

Fig 10 shows that the step response curves of each order transfer function model of the generator. The order of *tf*11 is lower and the step response speed is faster than *tf*12. Thus, the transfer function of the generator specimen is determined as *tf*11 in Tab.2 and is shown in Eq 9.


$$G_{p}(s)=\frac{2.817}{s+2.253}$$ (9)

### 3.3 Construction of transfer function model for wind power system

#### 3.3.1 Transfer function model of wind power system

If the wind rotor specimen is connected with the generator specimen, a wind power generation system is formed. According to Eq 7, the overall transfer function of the fictitious system can be obtained as shown in Eq 10. Although the wind rotor prototype is not connected to the generator prototype at present, we can use this global transfer function to evaluate the overall output characteristics of the system.


$$G(s)=G_{t}(s)\times G_{p}(s)=\frac{-7.208s+144.2}{s^{2}+8.657s+11.18}\times \frac{2.817}{s+2.253}$$ (10)

Since the order of the transfer function model of wind power system established by Eq 10 is relatively high, its mathematical operation is difficult to carry out. Therefore, the model is treated with reduced order after substituting the working parameters of each component.

#### 3.3.2 Reduced order of system transfer function model

**Fig 11:**
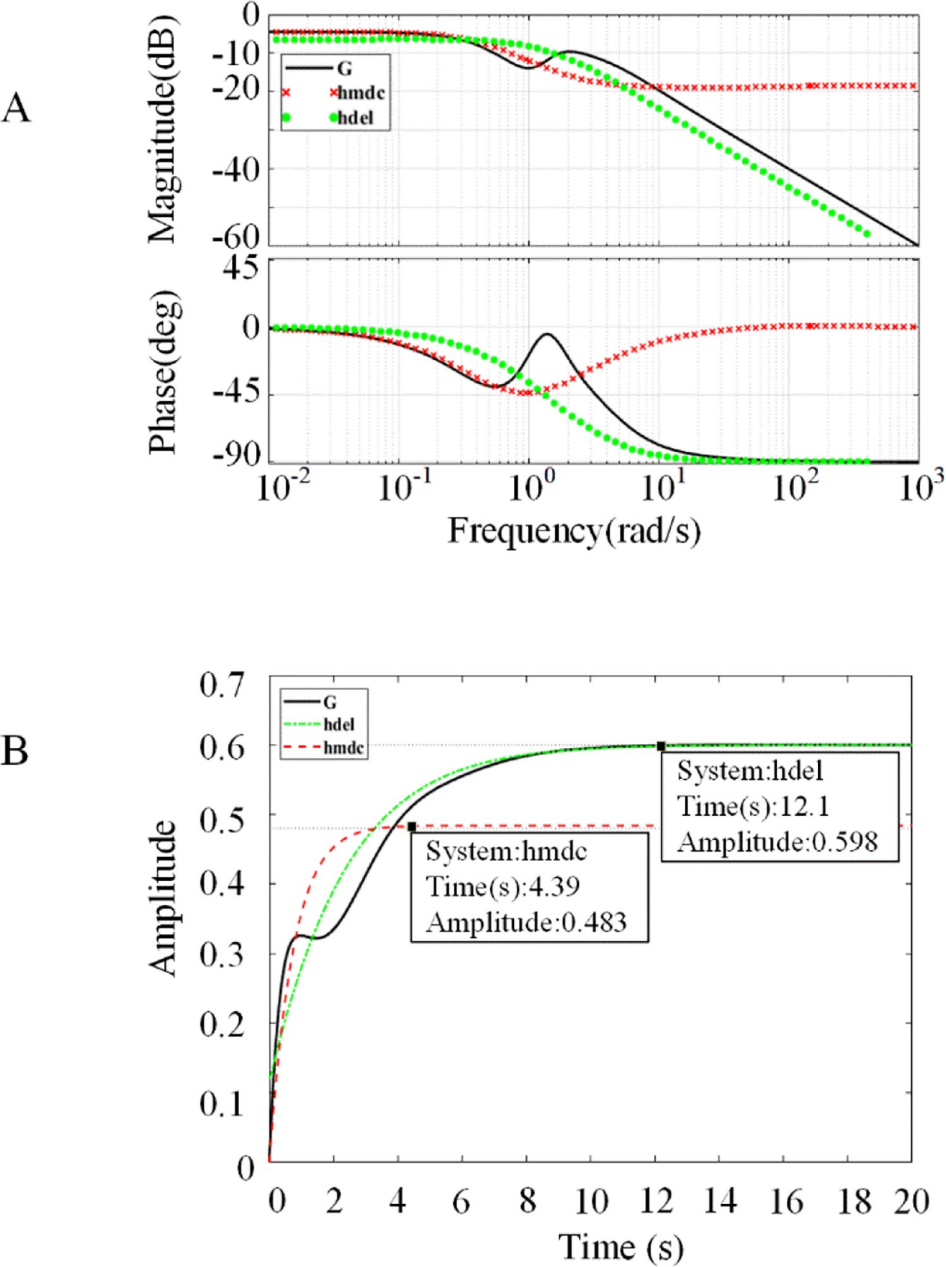
System reduction processing diagram. a) Amplitude-frequency characteristics of reduced order models. The hmdc model and hdel model are respectively compared with the amplitude-frequency characteristic curves of the original higher-order transfer function b) Reduced order model responses diagram. The hmdc model and hdel model are compared with the response curve of the original high order transfer function.

The transfer function of the system is processed by using two reduced order models of hmdc and hdel in MATLAB, and the amplitude-frequency characteristics are shown in Fig 11. In Fig 11A, the reduced order result of hdel model is close to the amplitude-frequency characteristic curve of the original higher-order transfer function, which can satisfy the curve characteristics of the original function well. Although the hmdc model can reflect the inflection point change of the original transfer function more accurately than the hdel model before 1rad/s, its curve gradually diverges compared to the original function when the frequency achieves 10rad/s. Fig 11B shows the response curves of the two models. The amplitude of hmdc model stabilized at 0.483 after 4.39s, however, the response of hdel model is close to the original transfer function, and the amplitude gradually converges to 0.598 after 12.1s. Therefore, the hdel model is selected as the reduced order model of the original higher-order transfer function of the system.

**Fig 12:**
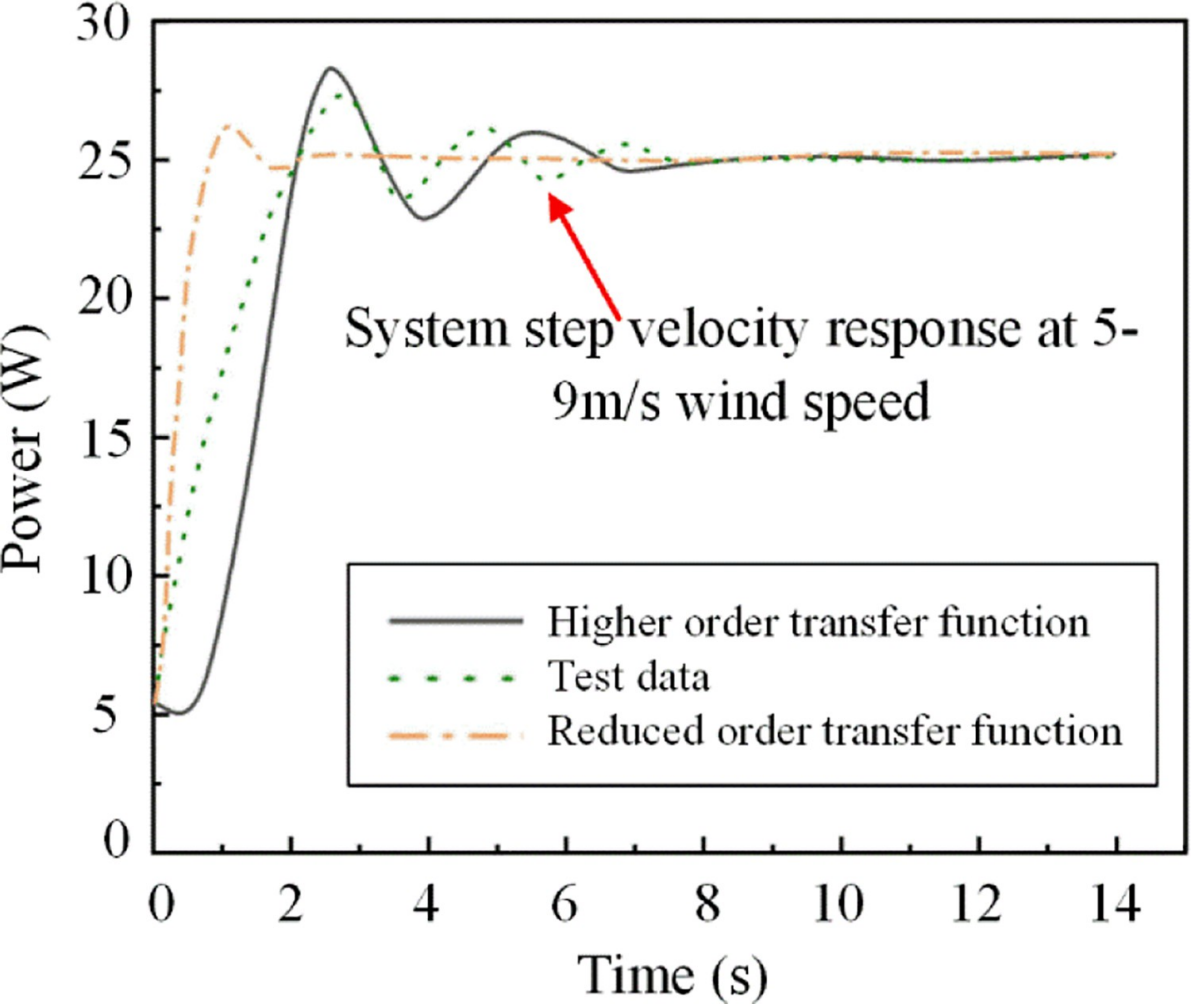
Speed response of step change of wind power system. The step velocity responses of the higher order model, the lower order model and the test are compared.

The simulation platform of wind power system is built in Simulink to simulate the speed response of the system under the wind speed of 5~9 m/s. The step velocity responses of the high-order model, the reduced-order model and the test are shown in Fig 12. The results show that the error of the velocity response curve of the reduced order model is 4.6% compared with that of the test curve, and the error of the high-order model curve is 3.1% compared with that of the test. Therefore, this reduced order model can be used to replace the higher-order model for analysis.

### 3.4 Analysis and verification of system transfer function model

#### 3.4.1 Wind tunnel test of the overall performance of wind power systems

**Fig 13:**
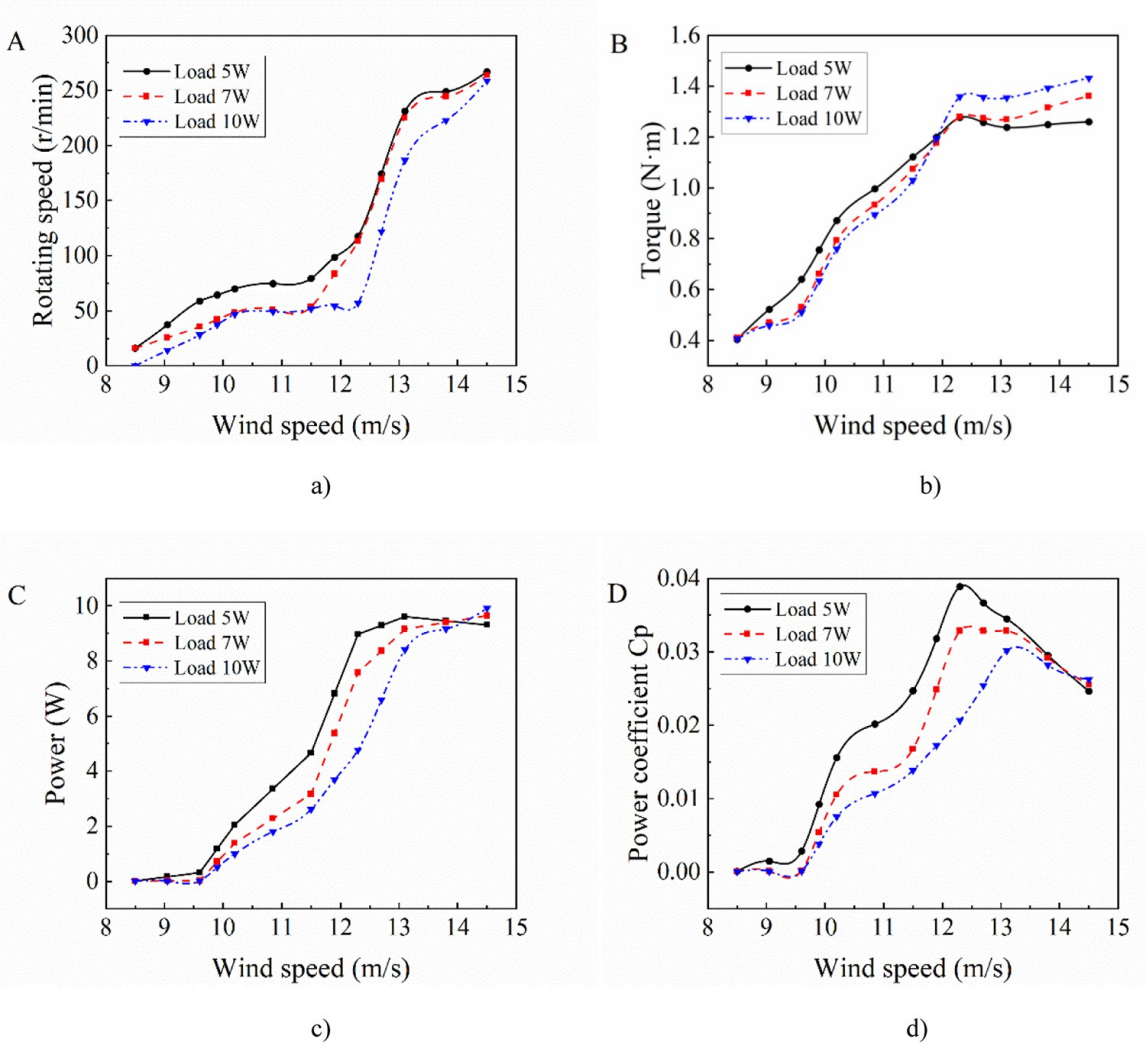
Output performance curves of the wind power system. a) Wind speed vs rotating speed. b) Wind speed vs torque. c) Wind speed vs power. d) Wind speed vs power coefficient.

The wind rotor and generator are connected on the test bench in Fig 5 to form a wind power system, and the overall output performance is obtained through wind tunnel tests. Fig 13 shows the characteristic curves of voltage, current, power and conversion efficiency respectively with the speed when the system loads are 5 W, 7 W and 10 W respectively. In Fig 13A, the speed of the wind rotor increases as the wind speed increases. With the increase of load, the speed of wind rotor at the same wind speed decreases. As shown in Fig 13B, the torque of the wind rotor increases with the increase of the wind speed. At the same wind speed condition, the torque of the wind rotor under higher load is bigger than that under lower load. Fig 13C shows that as the wind speed increases, the system output power increases. When the wind speed reaches the measured peak, the output power of wind rotor under higher load still shows a increasing trend. Fig 13D shows that although the output power of wind rotor increases with the increase of wind speed, the power coefficient increases firstly and then decreases, and there is a maximum efficiency point. In addition, with the increase of the load, the maximum power coefficient of the wind rotor decreases, indicating that performance of the generator driven by the wind rotor will affect that of the wind rotor.

#### 3.4.2 Data analysis

**Fig 14:**
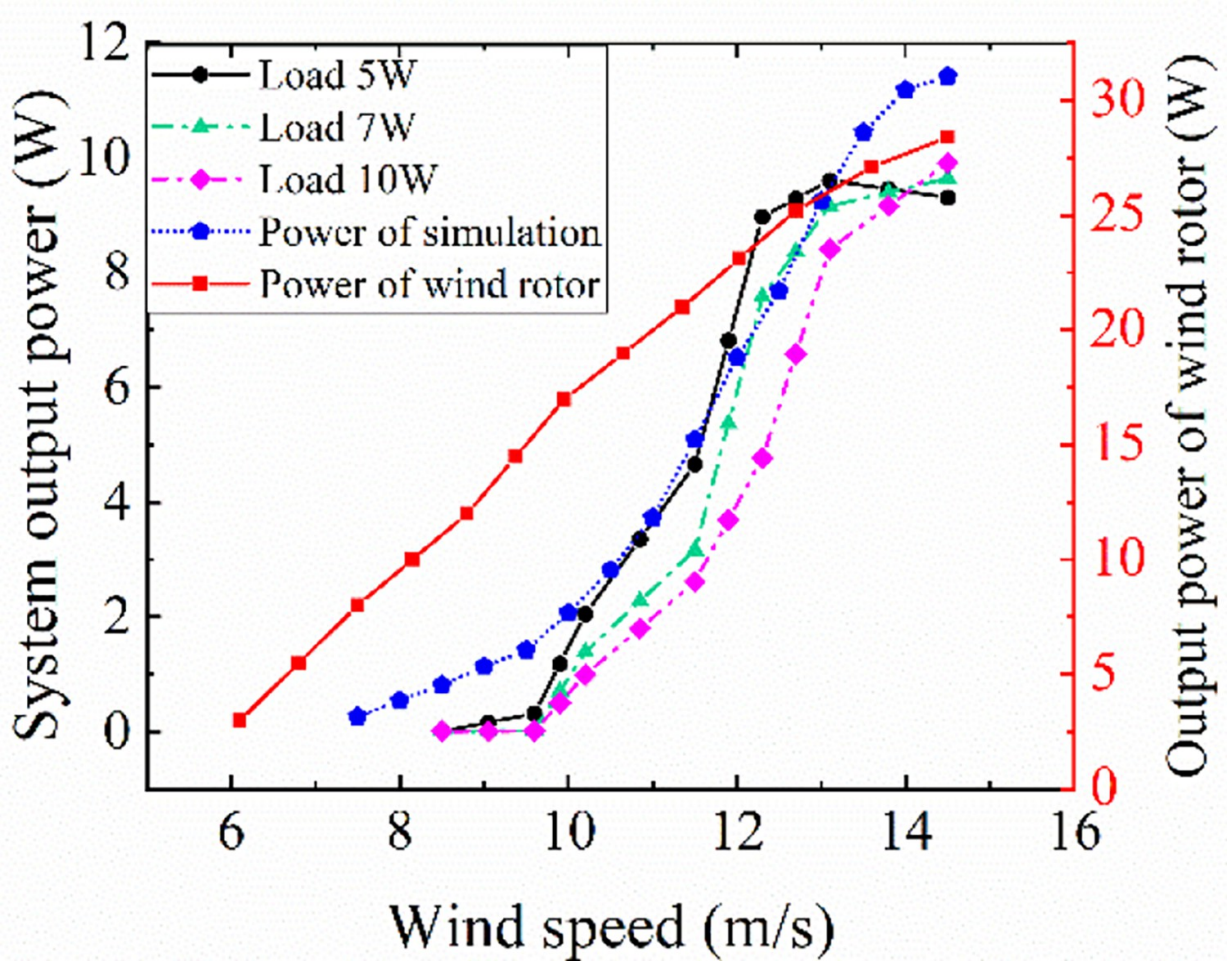
The output power diagram of three load tests, simulation models and wind rotor tests.

Fig 14 shows the output power curve of a single wind rotor, the overall output power curves of the wind power system by wind tunnel tests, and the simulation curve of the overall output power according to the reduced order transfer function of Eq 10.

As shown in Fig 14, the overall output power curve of the wind power system is far from that of the single wind rotor. When the wind speed is 14.5 m/s, the maximum output power of the wind rotor is 28.4 W, the output power of the system under three loads are 9.3 W, 9.6 W, and 9.9 W respectively, accounting for 32.7%, 33.8%, and 34.9% of the wind rotor output power. This indicates that when the wind rotor is connected to the generator to form the wind power system, the output power of the wind rotor decreases by 66.2%. Thus, the performance of the generator has an obvious influence on the wind rotor, and the overall output of the system is the result of the interaction of the two components. Therefore, the performance and matching relationship of the two components should be considered comprehensively in the system analysis. Errors between the simulation and the test data of the system is 4.1%, 3.6% and 6.5% respectively, and the variation trend of the simulation curve is consistent with that of the test curves. Thus, the simulation curve can accurately predict the output characteristics of the system power. This indicates that the transfer function model of wind power system can be predict the performance of the system.

When the wind rotor is connected to the generator, there is a direct relation between them through the speed. A simple assumption to estimate system output performance is that the product of the efficiency of the wind rotor and generator can be taken as the estimated efficiency of the overall output of the system. Therefore, the rotating speed is taken as the benchmark parameter to consider the overall output performance of the wind power system. Since wind speed is used as the dependent variable in the *C*_*p*_ curve of wind rotor and rotating speed in that of generator, the estimated efficiency curve of wind power system can be obtained by means of product two efficiencies at the same speed after the normalization of the dependent variables of the two components. The fitting equation between efficiency and rotating speed is obtained by fitting the generator efficiency curve with three polynomials, and the fitting efficiency equation of the generator is shown in Eq 11.


$$y=2.60\times 10^{-9}x^{3}-5.25\times 10^{-6}x^{2}+3.0\times 10^{-3}x+8.86\times 10^{-2}$$ (11)

**Fig 15:**
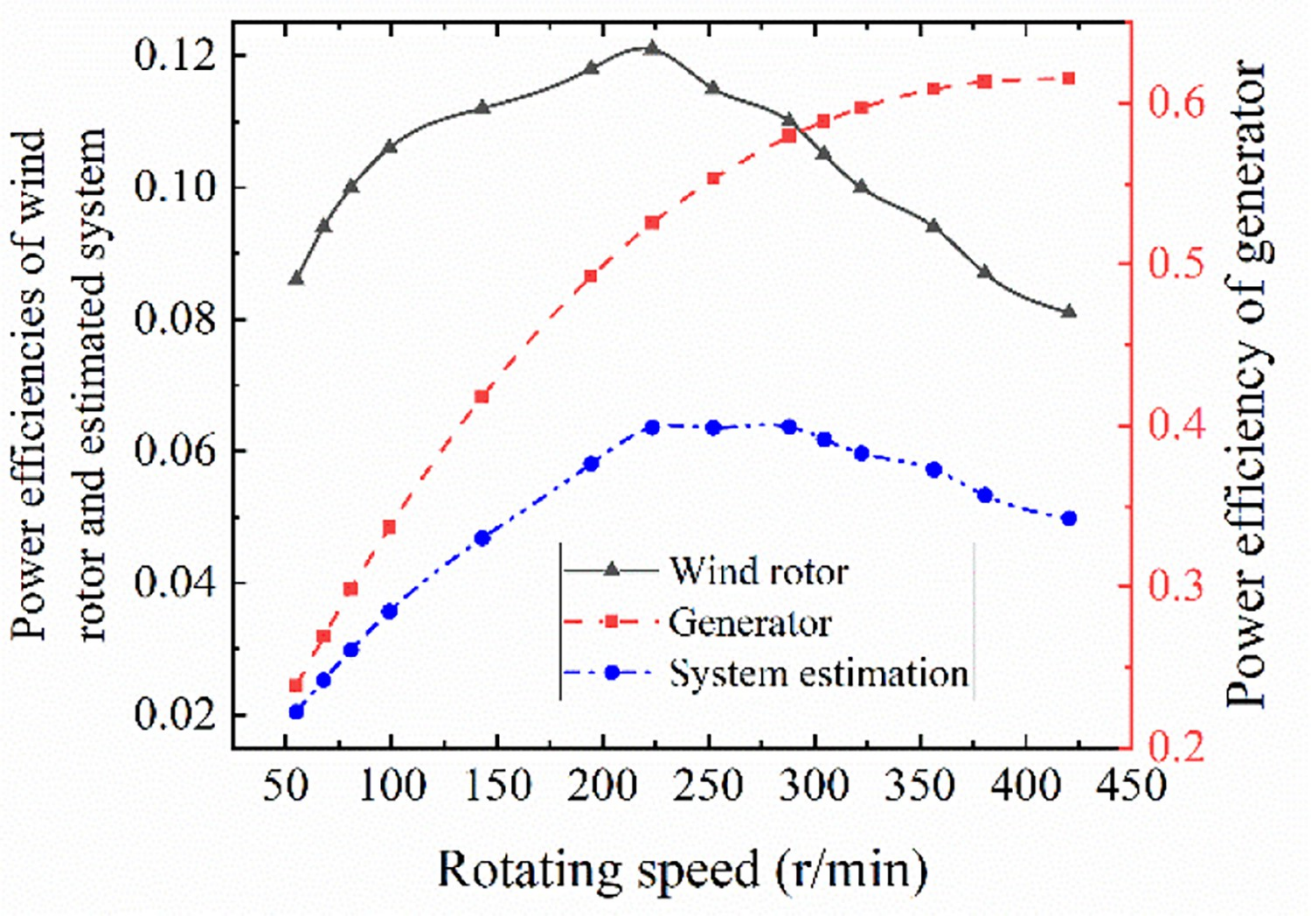
System estimated efficiency curve. The overall output efficiency is lower than that of a wind rotor or generator.

The rotating speed of the wind rotor is transferred to the generator, so that the rotating speed of the generator is the same as the former or a certain multiple by gear mechanism. Therefore, the rotating speed value of the wind rotor is substituted into Eq 11, and the efficiency of the motor under the same speed can be obtained. Then the generator efficiency curve with the rotating speed as the dependent variable is obtained. This method ensures the consistency of the dependent variables of the efficiency curves of the two components. The estimated efficiency curve of wind power system performance is obtained by multiplying the efficiencies of wind rotor and generator at the same speed, as shown in Fig 15. It shows that the overall output efficiency is lower than that of a wind rotor or generator. The maximum efficiency of the wind rotor is 12.1% when rotating speed is 223.3r/min, and the maximum efficiency of the system is 6.2% when rotation speed is 275r/min. The highest point of the system efficiency is obviously lower than and lags behind the wind rotor.

**Fig 16:**
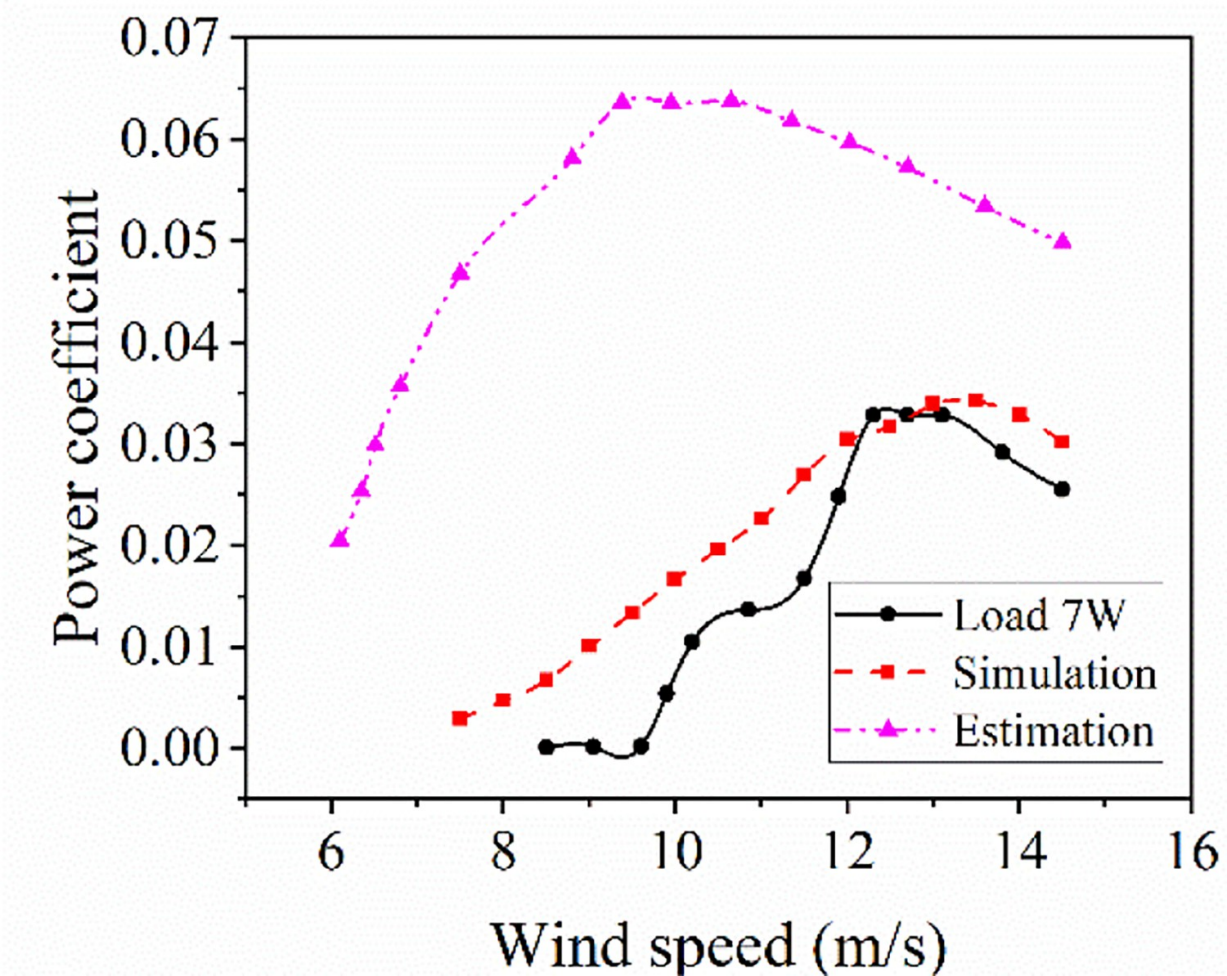
Comparison of the three efficiency curves of system. Comparison of the wind power system, the overall output efficiency curve of the system simulated by the transfer function model and the efficiency curve of the test under load 7 W.

The estimated overall output efficiency curve of the wind power system, the overall output efficiency curve of the system simulated by the transfer function model and the efficiency curve of the test under load 7 W are shown in Fig 16.

In Fig 16, test efficiency of the system reaches a maximum of 3.5% at wind speed of 12.3 m/s, and the maximum value of the predicted efficiency curve is 6.3% at wind speed of 9.8 m/s. The maximum value of the predicted efficiency is 80.0% higher than that of the test. Wind speed at the maximum efficiency point of the estimated efficiency curve reduces by 2.5 m/s, and the error is 20.3% than that of test. There is a big difference in the trend between the estimated and the test curve, this indicates that the overall output of the system by multiplying the efficiencies between wind rotor and generator cannot directly reflect the real operating characteristics of the system without considering the interaction of components. For simulation and test efficiency curves, the efficiency values corresponding to different wind speeds of the two curves are compared and the mean values are taken respectively, and the average error between the predicted and the test efficiency curve is 3.7%. The maximum efficiency value of the simulation curve is 3.4%, and the difference between it and the maximum efficiency value of the test is only 0.1%. It indicates that the simulation efficiency curve can reflect the output of the test better and the constructed transfer function model of wind power system can better predict the output characteristics of the system. It also shows that the performance matching between components within the wind power system can be explained and analyzed by the transfer function theory. In Reference 14, the coupling torque model of wind turbine and generator is established to investigate how to select the optimal blade diameter to match with the generator when the wind turbine can drive the optimized generator to start and rotate. Driving performance is the primary relationship considered between the two components. In Reference 15, a CFD simulation and analysis model of the wind wheel was established, and transient simulation calculation was carried out by turbulence model and coupling algorithm to obtain aerodynamic parameter curve of the two-dimensional CFD wind wheel model. On the basis of this method, the power characteristic curves and torque characteristic curves of the wind wheel under different wind speed conditions were obtained by changing the input wind speed. Then the matching characteristics of the wind rotor and the generator were analyzed by comparing with the given generator optimal power curve and torque curve and evaluating the coincidence degree of the curves. In this paper, by constructing and analyzing the component and system transfer functions of a wind rotor and a generator with known performance, the overall output performance characteristics of the wind power systems can be predicted. This method is helpful for the rapid selection of system components.

**Fig 17:**
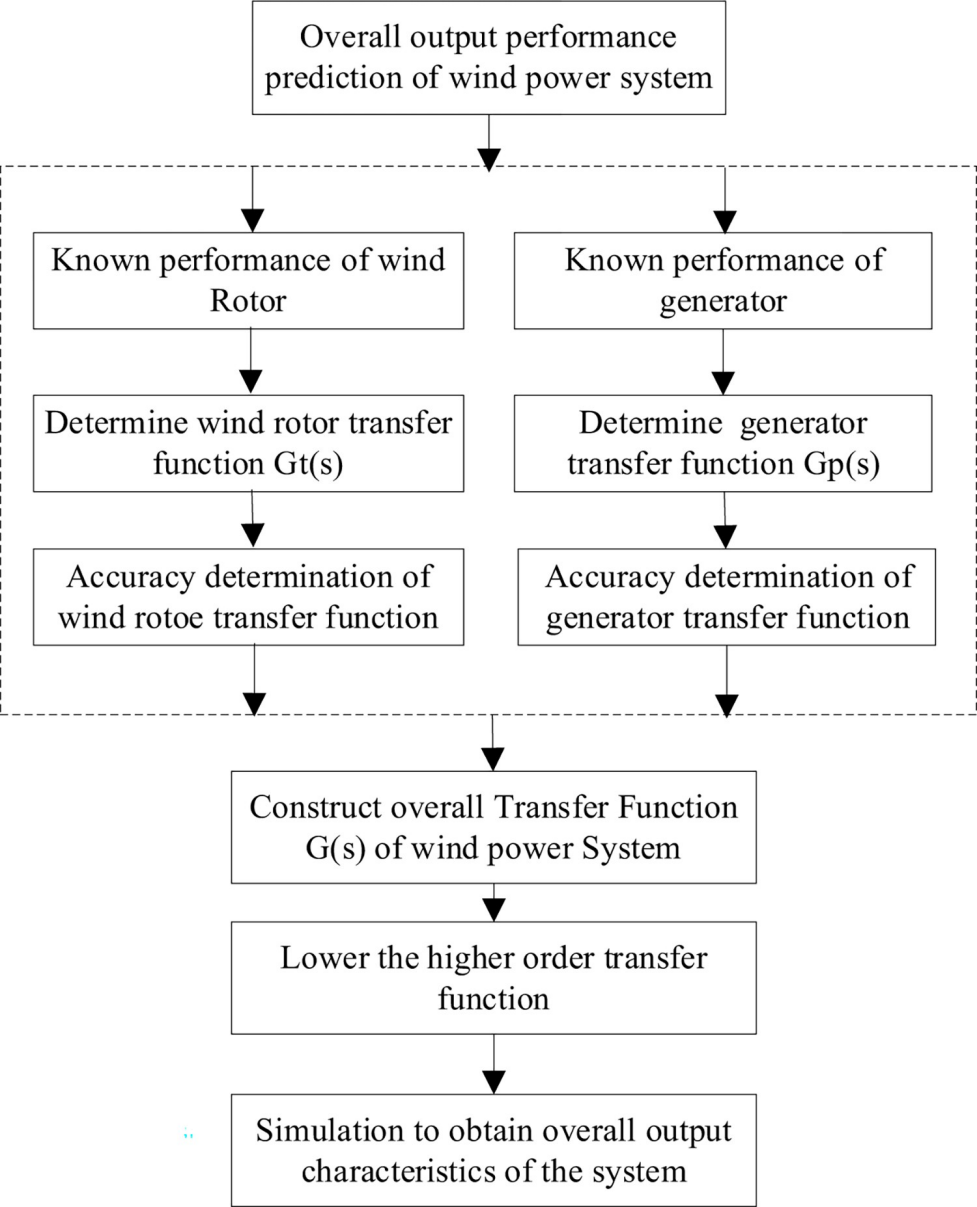
A short flowchart of the proposed method.

The short flowchart of the proposed method is shown in Fig 17. In order to predict the overall output characteristics of the wind power system, based on the known characteristics of wind rotor and generator, the transfer function expressions are determined respectively by using MATLAB system identification methods, and the accuracy of the transfer functions are determined. Then, the overall transfer function of the system is constructed. The whole output characteristics of the system are obtained by using the function simulation. This method can obtain the overall output characteristics of the wind power system without setting up the wind power system and carrying out tests, which greatly improves the design efficiency.

## 4. Concluding remarks

In this paper, a performance matching method of wind rotor and generator based on energy transfer is discussed.

A transfer function model of wind power system is established based on the structural characteristics of internal energy conversion in the system and transfer function theory. Simulation analysis for the transfer function model of the system overall output characteristics and comparation with wind tunnel test results are discussed. The results indicate that the proposed transfer function model can reflect the real output characteristics of wind power system. At the same time, the transfer function model is also better than the method considering efficiency product of wind rotor and generator as the overall output characteristics of the system. It shows that on the basis of the known performance parameters of wind rotor and generator, the overall transfer function model of wind power system can be established directly, and the overall output characteristics of the system can be analyzed and predicted directly, without the need to connect wind rotor and generator and test to obtain the system output characteristics.

The degree of matching between components within the system is an important factor affecting the overall output performance of the system. Compared with traditional methods, the proposed method can shorten design cycle and reduce development costs.

Some more in-depth thinking for the proposed matching approach may be considered:

In the process of component model construction on the premise of known component performance parameters, the proposed method constructs component transfer function model, and uses MATLAB to identify and determine the model parameters of transfer function. If different components are selected, the determination of the order and precision of the transfer function model sometimes depends on the experience of the designer due to their different performance parameters. This will lead to partial subjective uncertainty of the analysis results. How to construct a more objective parameter identification method will be one of the problems to be further considered. The identification and evaluation methods of the transfer function model parameters may be considered in the follow-up study.

In this study, only one wind rotor and one generator are selected respectively. In the follow-up study, the number of test pieces will be increased. Through multiple pairs of wind rotors and generators, cross-testing will be carried out to further verify the applicability of the model.

## Supporting information

S1 File (ZIP)
